# Time series analysis of the new tiering pricing policy for generic medicines in South Korea

**DOI:** 10.3389/fphar.2026.1763230

**Published:** 2026-03-31

**Authors:** Chan Mi Park, Inmyung Song, Dong-Sook Kim

**Affiliations:** 1 Biomedical Research Center, Korea University Guro Hospital, Seoul, Republic of Korea; 2 College of Nursing and Health, Kongju National University, Gongju, Republic of Korea

**Keywords:** generic, policy, pricing, South Korea, time series

## Abstract

**Background:**

South Korea introduced a new tiered pricing policy for generic medicines in July 2020, linking reimbursement to bioequivalence testing and active pharmaceutical ingredient registration requirements. While the policy aimed to improve market efficiency and quality assurance, its impact on product availability and generic uptake has not been fully evaluated.

**Methods:**

A retrospective observational study was conducted using the National Health Insurance Drug Master File and claims data from January 2017 to December 2022, with descriptive analyses covering 2017–2022. Interrupted time series (ITS) analysis with segmented regression was applied to assess changes in the number of marketed medicines and the expenditure-based market share of generics following the policy intervention, including stratified analyses by Anatomical Therapeutic Chemical level-1 categories. This study estimated policy effects using expenditure-based indicators under the revised reimbursement framework.

**Results:**

The policy resulted in a significant immediate increase in the number of generic medicines (+1,811; p < 0.001), followed by a reversal in trend from a pre-policy monthly increase to a post-policy decline. No significant immediate change was observed in the expenditure-based market share of generics; however, a significant positive post-policy trend was identified (+0.08 percentage points per month; p < 0.001).

**Conclusion:**

South Korea’s tiered pricing policy led to rapid structural adjustments in the generic medicines market, with substantial short-term increases in product registrations and gradual increases in generic expenditure share over time. By linking pricing to quality-related requirements, the policy may strengthen market competitiveness and quality compliance. Continued monitoring and class-specific complementary strategies are warranted to sustain long-term effectiveness.

## 1 Introduction

To curb rising pharmaceutical expenditures and promote sustainable competition in off-patent markets, many countries have implemented policies that encourage the use of generic medicines (Dylst and Simoens, 2010; 2011; Dylst, Vulto, Godman and Simoens, 2013; Simoens, 2008). A generic drug is defined as a product that matches the brand-name drug in dosage form, safety, strength, route of administration, quality, performance profile, and therapeutic use (FDA, 2021). Because approval of generics does not require repeating preclinical and clinical trials, these products are generally sold at substantially lower prices (Dylst and Simoens, 2010; 2011; FDA, 2021; Walker, 1995). The entry of generics following patent expiration may also prompt price reductions for the originator. Beyond generating budget savings, lower generic prices can enhance patient access and medication adherence by reducing out-of-pocket expenditures (Dylst and Simoens, 2010; 2011; Dylst et al., 2013; Kim, Shin and Chung, 2022; Simoens, 2008).

The economic gains from generic entry are considerable. In France, Germany, Italy, Spain, and the UK, the budgetary impact of exclusivity losses from biologics and small molecules is projected to reach nearly €23 billion by 2027, almost double the total from the previous 6 years (IQVIA, 2024). Across OECD countries, the average price of generic medicines fell by 43% in 2021 compared with 2007, with the United States showing the largest reduction at 68%. Most countries regulate generic prices through mechanisms such as internal and external reference pricing, tendering, discounts, tiered pricing, and payback systems (Dylst and Simoens, 2010; Francois et al., 2023; Puig-Junoy, 2012; Wouters and Kanavos, 2017). In response to recurring *ad hoc* price reductions, generic manufacturers have increasingly turned to contract manufacturing, contributing to growth in the global generic pharmaceuticals contract manufacturing market. This market was valued at USD 76.73 billion in 2024 and is projected to expand from USD 81.22 billion in 2025 to about USD 135.36 billion by 2034, with a CAGR of 5.84% during 2025–2034 (Generic Pharmaceuticals Contract Manufacturing Market, 2025).

In South Korea, from January 2012 to June 2020, both generic medicines and off-patent originator drugs were required to be priced at 53.55% of the on-patent originator’s price 1 year after generic entry (Kim et al., 2022). Since November 2010, the Pharmaceutical Affairs Act has permitted multiple market approvals based on previously submitted bioequivalence (BE) or clinical trial data, facilitating the proliferation of identical generics (MFDS, 2010). Prior to 2020, these policies largely relied on uniform pricing rules and repeated across-the-board price reductions, applying a single reimbursement level regardless of product-specific quality or regulatory characteristics.

This regulatory approach is considered to have contributed to the large-scale recall of 115 valsartan products on July 10, 2018 (H. Park, 2018). While earlier pricing reforms achieved short-term expenditure reductions, they were also criticized for encouraging excessive duplication of similar generic products and providing limited incentives for manufacturers to invest in quality assurance and regulatory compliance. To address these challenges, the government introduced a tiered pricing model for generics in July 2020, mandating both a new BE test and registration of the active pharmaceutical ingredient. Unlike previous uniform pricing approaches, the revised policy links reimbursement levels to the extent of regulatory compliance, thereby operationalizing a tiered pricing structure rather than applying a single uniform price cut. The underlying policy logic represents a shift from across-the-board price reductions toward a quality- and compliance-based pricing framework, intended to discourage excessive market entry of low-quality generics while maintaining sustainable competition. Unlike elasticity-based price discrimination commonly discussed in the health economics literature, Korea’s tiered pricing framework differentiates reimbursement levels according to regulatory compliance requirements (bioequivalence testing and API registration), rather than targeting distinct demand segments.

However, no prior studies have evaluated whether this differential pricing policy has reduced the number of marketed products or shifted the market share of generics. Accordingly, this study aims to analyze time-series trends in the number of marketed medicines and the market share of generics in South Korea from 2017 to 2022 following the introduction of the new tiered pricing policy.

## 2 Methods

### 2.1 New tiering pricing policy of generic medicines in South Korea

Under the new pricing policy, for generics entering the market before the twentieth generic entrant for a given substance, the price of the new generic is determined by how many of the required conditions are met. The price of generics satisfying both requirements -(1) conducting a new bioequivalence test for the product and (2) registering the active pharmaceutical ingredient (API) used in manufacturing-is set at 53.55% of the price of the originator product before generic entry, whereas those fulfilling only one or none of the requirements are priced at 45.52% and 38.69%, respectively. For generics entering the market after the twentieth generic entrant for a given substance, the price is set at 85% of the lowest reimbursed price. Overall, the tiered pricing model is intended to link reimbursement levels to regulatory compliance requirements, with an additional pricing rule applied after the twentieth entrant. The threshold of the twentieth entrant was determined by the Ministry of Health and Welfare based on historical market entry patterns in high-volume therapeutic areas, where price convergence was typically observed after approximately 15–20 generic entrants. The policy therefore aimed to differentiate early entrants based on regulatory compliance while preventing excessive market overcrowding beyond the twentieth entrant.

### 2.2 Study design and data

This study employed a retrospective observational design using national administrative datasets to evaluate the impact of South Korea’s 2020 tiered pricing policy for generic medicines. This study was reported in accordance with the Strengthening the Reporting of Observational Studies in Epidemiology (STROBE) statement for cohort studies. A time-series framework was applied to quantify longitudinal changes in the number of marketed medicines and the expenditure-based market share of generics during the pre-policy period (January 2017–June 2020) and the post-policy period (July 2020–December 2022). An interrupted time series (ITS) analysis with segmented regression was used to examine changes in level and trend following implementation of the policy in July 2020.

This study integrated three primary datasets: (1) the National Health Insurance (NHI) drug master file, (2) NHI claims data from the Health Insurance Review and Assessment Service (HIRA), and (3) the list of bioequivalent medicines provided by the Ministry of Food and Drug Safety (MFDS). The NHI system covers approximately 97% of the Korean population, and the claims data include all reimbursed outpatient and inpatient prescription records submitted to HIRA.

The NHI master file, a monthly reimbursed drug list, includes product-level characteristics such as generic and proprietary names, strength, formulation, route of administration, manufacturer, and maximum reimbursed price. Medicines were categorized based on identical active ingredients, strength, and route of administration. By cross-referencing the drug master file with the MFDS database using unique product codes, we identified whether it met the BE testing requirements.

We also used NHI claims data submitted by healthcare providers to the HIRA. The NHI system covers approximately 97% of the Korean population, and the claims data include all reimbursed outpatient and inpatient prescription records. These claims contain patients’ demographic characteristics, diagnosis codes, the international nonproprietary name (INN) of each drug, prescribed daily dose, and therapy duration. Claims were aggregated annually to calculate expenditure-based market shares for each therapeutic class (Chae, Kim and Kim, 2022; Lee, Park and Kim, 2021; D. Park, Lee and Kim, 2022).

We classified drug therapeutics according to the Anatomical Therapeutic Chemical (ATC) classification of the World Health Organization (WHOCC, 2024). For the purposes of this study, generic medicines were defined as all off-patent products listed in the reimbursement drug master file that shared the same active ingredient, strength, and route of administration as the originator product, regardless of whether they had fulfilled additional bioequivalence or API registration requirements. ATC codes were merged from HIRA records, and the route of administration was categorized as oral, injectable, or other.

### 2.3 Outcome measures and policy intervention

The main outcome measures were the number of marketed medicines and the market share of generic medicines based on expenditure. The monthly count of unique medicines was derived from the Drug Master File.

Medicines were grouped into single-source drugs, off-patent originator drugs, and generic medicines. Single-source drugs were defined as originator products with no approved bioequivalent competitors at the time of observation. Off-patent originator drugs referred to originator products whose patent protection had expired but which remained on the market. Generic medicines included all off-patent products approved after patent expiry that contained the same active ingredient, strength, and route of administration as the originator. Bioequivalence status was analyzed separately as a product characteristic. These three categories were mutually exclusive in all analyses. The market share was calculated using annual expenditure from NHI claims for each defined category. All market share measures in this study reflect expenditure-based shares rather than prescription volume. Expenditure-based market share was selected because reimbursement policies primarily operate through price regulation and reimbursement levels, making expenditure a more direct reflection of policy impact than prescription volume alone.

The study period spanned January 2017 to December 2022. The intervention point was set at July 2020, corresponding to the implementation of the tiered pricing model that modified reimbursement rules based on BE testing and active pharmaceutical ingredient (API) registration requirements.

### 2.4 Covariates and subgroup variables

We incorporated multiple product-level and substance-level characteristics into stratified analyses as follows: ATC level-1 category, route of administration (oral, injectable, other), year of entry into the NHI reimbursement list (pre-2007, 2007–June 2020, post-July 2020), number of suppliers per substance (1, 2–3, 4–19, ≥20), and manufacturer type (domestic, foreign-invested). The year 2007 was selected as a node time because it corresponds to the introduction of the Positive List System in South Korea, which marked a major shift in pharmaceutical reimbursement policy by requiring formal cost-effectiveness evaluation for coverage decisions.

### 2.5 Statistical analysis

To quantify changes in level and trend following the policy, we conducted an ITS analysis using segmented regression:

Y_t = β0 + β1·Time_t + β2·PostPolicy_t + β3·TimeAfterPolicy_t + ε_t.

Where β0 represents the baseline level, β1 the pre-policy trend, β2 the immediate level change following the intervention, and β3 the change in slope after policy implementation (post-policy trend change).

This interrupted time series design accounts for baseline outcome levels and pre-policy trends, thereby partially controlling for time-invariant confounding and gradual secular changes unrelated to the policy intervention.

Autocorrelation was assessed using Durbin–Watson statistics, and Newey–West standard errors were applied when serial correlation was detected. Statistical significance was defined as p < 0.05. ITS models were estimated separately for each ATC level-1 category (A–V). Parameter estimates for level and trend changes were standardized and used as inputs for agglomerative hierarchical clustering to identify therapeutic classes with similar response patterns. This clustering approach was intended to classify therapeutic classes according to similarity in model-estimated policy responses, rather than visual inspection of raw time-series trends.

As a robustness check, sensitivity analyses were conducted by re-estimating the ITS models after excluding the first 6 months following policy implementation to account for potential transitional effects.

All analyses were conducted using SAS version 9.4 (SAS Institute, Cary, NC) and R version 4.2.1 for clustering and visualization.

## 3 Results

### 3.1 Overall trends in the number of medicines and expenditures

As shown in Table 1, the total number of marketed medicines increased from 23,146 in 2017 to 26,334 in 2022. Generic medicines consistently accounted for the majority of marketed products, and their share increased from 80.2% in 2017 to 85.0% in 2022. In contrast, single-source products witnessed a contraction in market presence, declining at an average annual rate of 4.3%. Regarding administration routes, oral formulations demonstrated a steady upward trend (compound annual growth rate [CAGR]: 4.4%), whereas injectable and other forms declined by 3.1% and 1.1%, respectively. While total expenditures increased annually at a rate of 7.6%, the distribution varied, with injectable drugs showing the highest growth in spending (CAGR: 9.5%).

**TABLE 1:** Number of medicines and expenditures by market status of medicines.

Variables	2017		2018	2019	2020	2021	2022		Annual growth rate
No. of medicines	​	​	​	​	​	​	​	​	​
Total	23,146	(100)	24,274	24,825	27,503	27,091	26,334	(100)	2.6%
Market status of medicine	​	​	​	​	​	​	​	​	​
Single-source	3,946	(17.0)	3,970	3,331	3,306	3,225	3,165	(12.0)	−4.3%
Off-patent originators	634	(2.7)	651	734	721	763	778	(3.0)	4.2%
Generic medicines	18,566	(80.2)	19,653	20,760	23,476	23,103	22,391	(85.0)	3.8%
Administration	​	​	​	​	​	​	​	​	​
Oral drugs	16,361	(70.7)	17,278	18,551	20,922	20,780	20,299	(77.1)	4.4%
Injectable drugs	4,194	(18.1)	4,241	3,751	3,826	3,728	3,580	(13.6)	−3.1%
Dental or ointment	2,591	(11.2)	2,755	2,523	2,755	2,583	2,455	(9.3)	−1.1%
Expenditures (million USD)	​	​	​	​	​	​	​	​	​
Total	15,266	(100.0)	16,598	18,006	18,636	20,161	22,036	(100.0)	7.6%
Market status of medicine	​	​	​	​	​	​	​	​	​
Single-source	6,291	(41.2)	6,948	7,685	8,054	8,615	9,296	(42.2)	8.1%
Off-patent originators	751	(4.9)	633	792	758	890	1,034	(4.7)	6.6%
Generic medicines	8,224	(53.9)	9,017	9,529	9,824	10,655	11,706	(53.1)	7.3%
Administration	​	​	​	​	​	​	​	​	​
Oral drugs	10,942	(71.7)	11,833	12,714	13,143	14,146	15,354	(69.7)	7.0%
Injectable drugs	3,462	(22.7)	3,849	4,265	4,456	4,911	5,461	(24.8)	9.5%
Dental or ointment	863	(5.7)	916	1,027	1,036	1,104	1,222	(5.5)	7.2%

### 3.2 Drug characteristics before and after the tiered pricing policy

Table 2 summarizes medicine characteristics before and after implementation of the tiered pricing policy in July 2020. The total number of marketed medicines decreased from 30,425 in the pre-policy period to 29,049 in the post-policy period, while total pharmaceutical expenditures declined from USD 58,986 million to USD 51,716 million. This apparent reduction reflects a structural adjustment following policy implementation, rather than a long-term contraction relative to the 2017 baseline.

**TABLE 2:** Changes after the tiered pricing policy.

​	Number of medicines				Expenditure (million USD)			
	Before		After		Before		After	
Characteristics	No.	%	No.	%	Exp.	(% of total)	Exp.	(% of total)
Total	30,425	​	29,049	​	58,986	​	51,716	​
Time	​	​	​	​	​	​	​	​
Before 2007	4,575	(15.0)	3,754	(12.9)	15,517	(26.3)	10,419	(20.1)
2007–2020 (June)	25,850	(85.0)	21,749	(74.9)	43,469	(73.7)	39,417	(76.2)
After 2020 (July)	0	(0.0)	3,546	(12.2)	0	(0.0)	1,880	(3.6)
Administration	​	​	​	​	​	​	​	​
Oral drugs	22,292	(73.3)	22,276	(76.7)	41,898	(71.0)	36,234	(70.1)
Injectable drugs	4,795	(15.8)	3,947	(13.6)	13,770	(23.3)	12,632	(24.4)
Dental or ointment	3,338	(11.0)	2,826	(9.7)	3,318	(5.6)	2,850	(5.5)
ATC	​	​	​	​	​	​	​	​
A	4,411	(14.5)	4,514	(15.5)	9,092	(15.4)	8,093	(15.6)
B	1,889	(6.2)	1,750	(6.0)	5,855	(9.9)	4,966	(9.6)
C	5,121	(16.8)	5,414	(18.6)	10,843	(18.4)	10,061	(19.5)
D	1,156	(3.8)	893	(3.1)	602	(1.0)	558	(1.1)
G	963	(3.2)	1,053	(3.6)	1,783	(3.0)	1,562	(3.0)
H	339	(1.1)	325	(1.1)	550	(0.9)	538	(1.0)
J	4,080	(13.4)	3,664	(12.6)	6,622	(11.2)	4,273	(8.3)
L	1,054	(3.5)	930	(3.2)	6,789	(11.5)	7,001	(13.5)
M	2,451	(8.1)	2,200	(7.6)	3,528	(6.0)	3,062	(5.9)
N	3,900	(12.8)	3,887	(13.4)	6,522	(11.1)	5,978	(11.6)
P	54	(0.2)	42	(0.1)	40	(0.1)	29	(0.1)
R	2,425	(8.0)	2,153	(7.4)	2,736	(4.6)	1,945	(3.8)
S	1,683	(5.5)	1,545	(5.3)	2,034	(3.4)	1,956	(3.8)
V	899	(3.0)	679	(2.3)	1,989	(3.4)	1,694	(3.3)
Number of suppliers	​	​	​	​	​	​	​	​
1	3,284	(10.8)	2,785	(9.6)	15,286	(25.9)	15,010	(29.0)
2–3	2,931	(9.6)	2,416	(8.3)	7,056	(12.0)	5,707	(11.0)
4–19	6,571	(21.6)	6,171	(21.2)	10,938	(18.5)	9,121	(17.6)
≥20	17,639	(58.0)	17,677	(60.9)	25,706	(43.6)	21,878	(42.3)
Classification of companies	​	​	​	​	​	​	​	​
Foreign-invested company	2,186	(7.2)	1,957	(6.7)	19,299	(32.7)	17,070	(33.0)
Domestic company	28,239	(92.8)	27,092	(93.3)	39,687	(67.3)	34,646	(67.0)
Distribution of reimbursement price	​	​	​	​	​	​	​	​
Q1	2,283	(7.5)	2,254	(7.8)	27	(0.0)	77	(0.1)
Q2	2,614	(8.6)	2,557	(8.8)	733	(1.2)	867	(1.7)
Q3	4,595	(15.1)	4,261	(14.7)	4,502	(7.6)	4,444	(8.6)
Q4	20,933	(68.8)	19,977	(68.8)	53,724	(91.1)	46,328	(89.6)
Bioequivalence	​	​	​	​	​	​	​	​
Yes	7,619	(25.0)	8,474	(29.2)	12,335	(20.9)	10,867	(21.0)
No	23,104	(75.9)	20,816	(71.7)	46,651	(79.1)	40,849	(79.0)

A (alimentary tract and metabolism), B (blood and blood forming organs), C (cardiovascular system), D (dermatologicals), G (genito-urinary system and sex hormones), H (systemic hormonal preparations, excl. Sex hormones and insulins), J (antiinfectives for systemic use), L (antineoplastic and immunomodulating agents), M (musculo-skeletal system), N (nervous system), P (antiparasitic products, insecticides and repellents), R (respiratory system), S (sensory organs), V (various).

By route of administration, oral medicines accounted for the majority of marketed products in both periods, with little change in absolute number, whereas injectable medicines saw a more pronounced decline from 4,795 to 3,947. Notably, expenditures for both oral and injectable formulations followed a downward trend.

Across ATC level-1 categories, changes in the number of marketed products varied by therapeutic class. The number of products in class A (alimentary tract and metabolism) rose slightly from 4,411 before the policy to 4,514 after implementation, and class C (cardiovascular system) increased from 5,121 to 5,414. In contrast, several other classes, including B (blood and blood forming organs), J (antiinfectives for systemic use), and R (respiratory system medicines), experienced reductions in product counts. Despite these differences, pharmaceutical expenditures declined across most ATC categories, including classes A and C.

Medicines supplied by 20 or more manufacturers remained the largest group in both periods, with their proportion slightly increasing from 58.0% to 60.9%. Regarding company classification, both foreign-invested and domestic companies saw a reduction in the number of their marketed products following the policy implementation.

The number of bioequivalent medicines rose from 7,619 in the pre-policy period to 8,474 in the post-policy period. However, despite this increase in product count, the corresponding expenditures for bioequivalent medicines declined from USD 12,335 million to USD 10,867 million during the same timeframe.

### 3.3 Impact of the tiered pricing policy: Interrupted time series analysis

**FIGURE 1:**
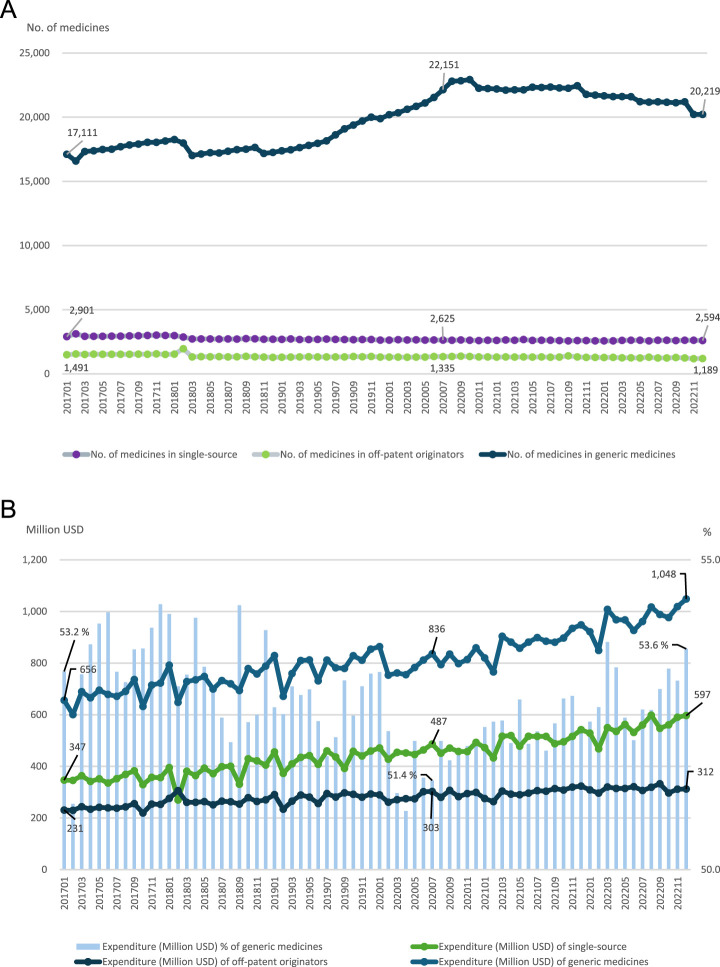
Trend in the number of medicines and expenditure after the new policy of generic medicines.

As shown in Figure 1, the interrupted time series (ITS) analysis identified a significant immediate increase in the number of generic medicines at the time of policy implementation (level change: +1,811; p < 0.001). Prior to the policy, the number of generic medicines exhibited a significant upward trend (+102.5 per month; p < 0.001), which shifted to a significant downward trend after implementation (−147.0 per month; p < 0.001). Subsequent trend estimates showed a gradual contraction after the initial post-policy period. Sensitivity analyses excluding the first 6 months after policy implementation, by re-estimating the ITS models, yielded qualitatively similar results, indicating that the main findings were robust to the choice of adjustment period.

**FIGURE 2:**
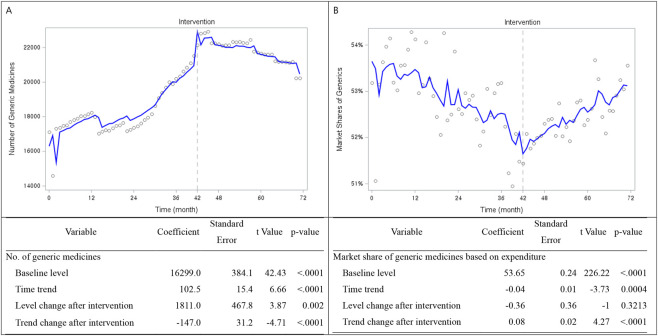
Interrupted time series of changes in the number of generic medicines and market share.

In contrast, Figure 2 presents the ITS results for the expenditure-based market share of generic medicines. The baseline market share was 53.65% (p < 0.001), with a small but significant declining pre-policy trend (−0.04 percentage points per month; p < 0.001). No significant immediate change was observed at the time of policy implementation (−0.36 percentage points; p = 0.32); however, the post-policy trend shifted in a positive direction, with generic market share increasing by 0.08 percentage points per month (p < 0.001). Specifically, while the number of generic medicines declined after the initial post-policy period, the expenditure-based market share of generics increased steadily over time. Because market share was measured on an expenditure basis, observed increases may reflect changes in price structure, product mix, or utilization, rather than volume effects alone.

### 3.4 ATC-level differences in policy impact

Interrupted time series estimates revealed substantial heterogeneity across all 14 ATC level-1 categories with respect to post-policy level and trend changes (Table 3). Significant positive post-policy level changes in the number of generic medicines were observed in several high-volume categories, including class C (cardiovascular: +382.6, p < 0.01) and class N (nervous system: +305.6, p < 0.001); however, both categories also demonstrated significant negative trend changes, indicating an initial spike followed by stabilization or decline. With respect to expenditure-based generic market share, significant positive post-policy trend changes were identified for class G (genito-urinary: +0.39% per month, p < 0.001) and class L (antineoplastic and immunomodulating agents: +0.18% per month, p < 0.001). Conversely, class J (antiinfectives for systemic use) showed a significant negative trend change (−0.20% per month; p < 0.001), while respiratory system drugs (R) showed a positive but statistically insignificant post-policy trend.

**TABLE 3:** Parameter estimates from interrupted time series analysis after policy by ATC level-1.

​	Number of generic medicines		Market share of generic medicines based on expenditure	
Variable	Coefficient	Standard error	Coefficient	Standard error
A (alimentary tract and metabolism)	​	​	​	​
Baseline level	2483.0^***^	47.1	45.78^***^	0.48
Time trend	19.3^***^	1.9	−0.06^**^	0.02
Level change after intervention	301.4^***^	62.0	−0.74	0.72
Trend change after intervention	−27.9^***^	3.8	0.10^*^	0.04
B (blood and blood forming organs)	​	​	​	​
Baseline level	890.0^***^	42.1	50.43^***^	0.49
Time trend	1.8	1.6	−0.07^***^	0.02
Level change after intervention	60.2	32.5	1.49^*^	0.68
Trend change after intervention	−0.2	3.3	0.17^***^	0.04
C (cardiovascular system)	​	​	​	​
Baseline level	2956.0^***^	84.6	56.12^***^	0.31
Time trend	21.4^***^	3.4	−0.02	0.01
Level change after intervention	382.6^***^	109.8	0.46	0.48
Trend change after intervention	−16.5^*^	6.9	0.06^*^	0.03
D (dermatologicals)	​	​	​	​
Baseline level	560.9^***^	15.8	45.34^***^	1.24
Time trend	−1.6^*^	0.6	0.16^**^	0.05
Level change after intervention	73.8^***^	20.4	−1.16	1.54
Trend change after intervention	−1.9	1.3	−0.68^***^	0.10
G (genito-urinary system and sex hormones)			​	​
Baseline level	421.1^***^	11.7	39.50^***^	0.60
Time trend	7.7^***^	0.5	0.01	0.03
Level change after intervention	103.3^***^	12.4	−0.59	0.93
Trend change after intervention	−10.8^***^	0.9	0.39^***^	0.05
H (systemic hormonal preparations, excl. Sex hormones and insulins)				​
Baseline level	176.1^***^	4.6	52.23^***^	0.71
Time trend	0.4^*^	0.2	−0.32^***^	0.03
Level change after intervention	5.5	3.6	3.53^**^	1.12
Trend change after intervention	−0.1	0.4	0.14^*^	0.06
J (antiinfectives for systemic use)	​	​	​	​
Baseline level	2427.0^***^	52.6	61.91^***^	0.62
Time trend	14.1^***^	2.1	0.23^***^	0.03
Level change after intervention	205.7^**^	63.5	−2.53^*^	0.98
Trend change after intervention	−27.7^***^	4.3	−0.20^***^	0.05
L (antineoplastic and immunomodulating agents)		​	​	​
Baseline level	529.6^***^	6.1	39.46^***^	0.46
Time trend	−1.9^***^	0.2	−0.27^***^	0.02
Level change after intervention	13.3	7.4	1.59^*^	0.72
Trend change after intervention	1.3^**^	0.5	0.18^***^	0.04
M (musculo-skeletal system)	​	​	​	​
Baseline level	1586.0^***^	43.9	71.93^***^	0.79
Time trend	3.3	1.8	−0.21^***^	0.03
Level change after intervention	130.9^*^	51.6	−2.07	1.12
Trend change after intervention	−6.5	3.6	0.16^*^	0.06
N (nervous system)	​	​	​	​
Baseline level	1905.0^***^	53.0	59.63^***^	0.46
Time trend	21.2^***^	2.1	0.16^***^	0.02
Level change after intervention	305.6^***^	64.8	1.35^*^	0.67
Trend change after intervention	−26.9^***^	4.3	0.06	0.04
P (antiparasitic products, insecticides and repellents)		​	​	​
Baseline level	23.1^***^	0.9	59.35^***^	2.04
Time trend	0.0	0.0	0.09	0.09
Level change after intervention	2.4^*^	1.2	−1.20	3.25
Trend change after intervention	−0.1	0.1	−0.11	0.17
R (respiratory system)	​	​	​	​
Baseline level	1282.0^***^	33.1	56.88^***^	1.01
Time trend	9.1^***^	1.3	0.08	0.04
Level change after intervention	112.1^**^	40.5	−3.66^*^	1.46
Trend change after intervention	−14.4^***^	2.7	0.10	0.08
S (sensory organs)	​	​	​	​
Baseline level	643.6^***^	13.9	56.36^***^	0.56
Time trend	9.2^***^	0.6	0.11^***^	0.02
Level change after intervention	157.4^***^	20.9	0.54	0.83
Trend change after intervention	−16.3^***^	1.1	−0.05	0.05
V (various)	​	​	​	​
Baseline level	424.0^***^	4.7	49.53^***^	0.49
Time trend	−2.1^***^	0.2	−0.01	0.02
Level change after intervention	11.0	6.4	0.26	0.73
Trend change after intervention	0.0	0.4	0.05	0.04

*p < .05, **p < .01, ***p < .001.

Cluster analysis using standardized interrupted time series (ITS) level and post-policy trend coefficients grouped ATC categories based on similarity in their response profiles (Supplementary Figure S2). Most therapeutic classes, including A, B, C, M, N, S, L, and G, were positioned near zero or modest level and trend changes. In contrast, classes J and R were characterized by negative level and post-policy trend changes, while class D appeared as an isolated category. These groupings reflect similarity in model-derived parameter estimates rather than visually distinct clustering patterns, indicating heterogeneity in policy responses across therapeutic areas.

## 4 Discussion

This study evaluated the impact of South Korea’s 2020 tiered pricing policy on the generic medicines market using national administrative datasets and interrupted time series analysis. The policy was associated with a substantial immediate increase in the number of generic medicines, followed by a significant decline in the post-policy trend. This pattern suggests that manufacturers initially accelerated product registrations to satisfy the strengthened BE and API requirements before adapting to a more regulated competitive environment. Such transitional dynamics are consistent with international experiences in which quality-linked pricing reforms prompt short-term strategic adjustments that are later followed by market stabilization (Dylst and Simoens, 2010; 2011; Dylst et al., 2013; Francois et al., 2023). Notably, although the post-policy decline in the number of generic medicines was statistically significant, the total number remained above the baseline level observed in 2017, suggesting that the policy did not reduce generic availability below historical levels. Descriptive comparison of product characteristics before and after policy implementation indicates that market exits were more concentrated among non-bioequivalent products, whereas the number of BE-certified generics continued to increase post-policy. This pattern suggests that the observed post-policy contraction reflects quality-driven consolidation rather than a reduction in the availability of compliant generic products.

In contrast to these pronounced structural changes, shifts in expenditure-based market share were more gradual. Although no abrupt change occurred at the time of policy implementation, a modest upward trend subsequently emerged, indicating slow but steady expansion in generic uptake. The reversal in trend direction may reflect changes in market composition induced by the policy, whereby products failing to meet strengthened regulatory requirements were gradually phased out, while remaining generics were perceived as more reliable substitutes for originators. In particular, the observed increase in the availability of bioequivalent products suggests that the policy effectively incentivized manufacturers to invest in quality assurance to maintain higher reimbursement levels. The steady increase in the number of bioequivalent medicines observed in Supplementary Figure S3 further supports the interpretation that the tiered pricing policy successfully incentivized higher regulatory compliance rather than reducing overall generic supply. In this context, the positive post-policy trend is likely driven not by an immediate increase in prescribing volume, but by incremental changes in prescriber confidence and substitution behavior as the market adjusted to a smaller but higher-compliance pool of generic medicines. Importantly, increases in expenditure-based generic market share should not be interpreted as a direct proxy for prescribing volume, as reimbursement-tier adjustments and price differentiation may independently influence spending patterns without corresponding changes in utilization. Prior Korean studies similarly report that supply-side effects of pricing reforms materialize more rapidly than downstream changes in prescriber or patient behavior, with utilization adapting only incrementally over time (Kim et al., 2022). The relatively modest magnitude of change may also reflect Korea’s already high baseline penetration of generics, which limits the potential for dramatic increases.

Substantial heterogeneity was observed across therapeutic categories. High-volume areas—such as the alimentary tract and metabolism, cardiovascular, and nervous system medicines—displayed strong immediate increases in the number of generic products. In contrast, antibiotics showed increased product availability but declining expenditure share, a pattern likely driven by antimicrobial stewardship initiatives and clinical appropriateness constraints that restrict economic substitution. Descriptive patterns by manufacturer type suggest that post-policy structural changes may have been more pronounced in markets dominated by domestic manufacturers, whereas segments with a higher presence of foreign-invested manufacturers exhibited more stable product counts. Although formal inferential analyses by reimbursement price quartiles were not conducted, descriptive patterns indicate that post-policy consolidation was more evident among higher-priced medicines. Cluster analysis further demonstrated that therapeutic classes respond differently to the pricing reform, underscoring that generic pricing policies do not operate uniformly across clinical domains and may require complementary, class-specific interventions (Dylst et al., 2013; Francois et al., 2023).

The findings align with earlier Korean evaluations of price-centered reforms, including the 2012 large-scale price cuts, which substantially reduced expenditures but produced limited or gradual shifts in prescription volume, with notable variation across therapeutic classes (Kwon and Godman, 2016). Similar patterns have been documented for antidiabetic and antihypertensive drugs, where utilization remained relatively stable despite reductions in cost (IQVIA, 2024). Compared with these earlier reforms, the current study examines a policy that explicitly links reimbursement to regulatory compliance—rather than price alone—thereby offering new insight into how quality-linked pricing incentives shape product availability and competitive behavior. International evidence from China’s procurement reforms and South Africa’s single exit price policy similarly highlights immediate structural effects accompanied by heterogeneous therapeutic-class outcomes (Moodley and Suleman, 2019; Rong, Yin, Duan, Sun and Babar, 2020). The present study contributes to this literature by providing detailed ATC-level evaluation and clustering of response patterns.

This study contributes to the evidence base in three important ways. First, it provides an early empirical assessment of a quality-linked tiered pricing policy, a framework increasingly discussed but seldom evaluated in practice. Second, by integrating product-level supply indicators with expenditure-based demand outcomes, the study offers a balanced perspective on both structural and utilization-related effects, extending prior Korean work that focused predominantly on expenditures. Third, its systematic assessment of therapeutic-class heterogeneity and cluster-derived response patterns yields actionable insights for designing differentiated policy strategies tailored to clinical context and market conditions.

This study has several limitations. First, reliance on aggregated administrative data prevented the evaluation of prescriber-level decision-making, patient-level switching, or adherence behaviors. Second, although the interrupted time series design accounts for baseline levels and pre-policy trends, unmeasured contemporaneous factors—such as antimicrobial stewardship initiatives, enhanced regulatory inspections, changes in clinical guidelines, and broader healthcare system dynamics—may have influenced the observed trends during the study period. These factors may bias estimates of the policy effect in either direction. Third, contemporaneous regulatory and clinical initiatives—such as enhanced quality inspections and antibiotic stewardship policies—may have contributed to observed therapeutic-class patterns. Finally, expenditure-based market share does not fully capture prescribing volume; future analyses including prescription counts or patient-level claims would allow more nuanced characterization of behavioral responses. Although the findings are specific to the Korean pharmaceutical reimbursement system, the results may be informative for other countries considering quality-linked pricing reforms in highly regulated generic markets.

## 5 Conclusion

Overall, the 2020 tiered pricing policy produced immediate structural adjustments in Korea’s generic medicines market and a gradual upward trend in expenditure-based generic use. By linking reimbursement to BE and API compliance, the policy appears to strengthen the quality orientation and competitive structure of the generic market. Continued monitoring, along with complementary efforts such as prescriber education, transparency in regulatory information, and improved patient communication, will be essential to sustain these gains and ensure that market competition remains both efficient and clinically appropriate.

## Data Availability

The raw data supporting the conclusions of this article will be made available by the authors, without undue reservation.
