# Supplementary material for: Time series analysis of the new tiering pricing policy for generic medicines in South Korea
Source: Front Pharmacol. 2026 Mar 31;17:1763230. doi: 10.3389/fphar.2026.1763230 (PMC13076297; doi:10.3389/fphar.2026.1763230)
Supplement: Supplementary file 1 [file Supplementaryfile1.docx]

Supplementary Table 1. Number of medicines and expenditure by ATC level-1

|  | No. of medicines | | | | | | | Expenditure (Million USD) | | | | | | |
| --- | --- | --- | --- | --- | --- | --- | --- | --- | --- | --- | --- | --- | --- | --- |
|  | 2017 | 2018 | 2019 | 2020 | 2021 | 2022 | (AGR) | 2017 | 2018 | 2019 | 2020 | 2021 | 2022 | AGR |
| A (alimentary tract and metabolism) | | | | | | | |  |  |  |  |  |  |  |
| Single-source | 445 | 463 | 408 | 411 | 401 | 411 | (-1.6) | 1,195 | 1,274 | 1,402 | 1,479 | 1,605 | 1,736 | (7.8) |
| Off-patent originators | 57 | 57 | 54 | 57 | 63 | 60 | (1.0) | 106 | 120 | 133 | 139 | 182 | 202 | (13.9) |
| Generic medicines | 2,766 | 2,987 | 3,282 | 3,735 | 3,618 | 3,661 | (5.8) | 1,084 | 1,165 | 1,221 | 1,271 | 1,370 | 1,500 | (6.7) |
| B (blood and blood forming organs) | | | | | | | |  |  |  |  |  |  |  |
| Single-source | 362 | 365 | 317 | 312 | 305 | 285 | (-4.7) | 667 | 719 | 793 | 822 | 807 | 812 | (4.0) |
| Off-patent originators | 102 | 111 | 123 | 131 | 136 | 148 | (7.7) | 75 | 77 | 129 | 127 | 130 | 192 | (20.6) |
| Generic medicines | 1,011 | 1,044 | 988 | 1,130 | 1,235 | 1,125 | (2.2) | 774 | 852 | 856 | 903 | 1,035 | 1,051 | (6.3) |
| C (cardiovascular system) | | | | | | | |  |  |  |  |  |  |  |
| Single-source | 406 | 407 | 405 | 421 | 429 | 445 | (1.9) | 1,186 | 1,293 | 1,438 | 1,550 | 1,689 | 1,781 | (8.5) |
| Off-patent originators | 44 | 49 | 51 | 48 | 49 | 55 | (4.6) | 28 | 33 | 38 | 36 | 36 | 50 | (12.1) |
| Generic medicines | 3,375 | 3,504 | 3,855 | 4,448 | 4,597 | 4,509 | (6.0) | 1,580 | 1,696 | 1,813 | 2,004 | 2,226 | 2,427 | (9.0) |
| D (dermatologicals) | | | | | | | |  |  |  |  |  |  |  |
| Single-source | 358 | 343 | 237 | 236 | 227 | 210 | (-10.1) | 79 | 76 | 70 | 83 | 108 | 126 | (9.7) |
| Off-patent originators | 19 | 20 | 34 | 32 | 32 | 39 | (15.5) | 9 | 10 | 11 | 10 | 13 | 15 | (10.2) |
| Generic medicines | 604 | 611 | 564 | 605 | 557 | 516 | (-3.1) | 81 | 84 | 92 | 97 | 97 | 98 | (3.9) |
| G (genito-urinary system and sex hormones) | | | | | | | |  |  |  |  |  |  |  |
| Single-source | 90 | 90 | 79 | 83 | 85 | 81 | (-2.1) | 238 | 274 | 296 | 301 | 307 | 280 | (3.3) |
| Off-patent originators | 42 | 42 | 38 | 36 | 31 | 28 | (-7.8) | 30 | 33 | 35 | 38 | 47 | 33 | (2.0) |
| Generic medicines | 551 | 618 | 705 | 893 | 836 | 831 | (8.6) | 182 | 198 | 217 | 240 | 280 | 316 | (11.7) |
| H (systemic hormonal preparations, excl. sex hormones and insulins) | | | | | | | | |  |  |  |  |  |  |
| Single-source | 71 | 72 | 68 | 69 | 74 | 72 | (0.3) | 46 | 54 | 69 | 82 | 94 | 120 | (21.0) |
| Off-patent originators | 17 | 17 | 19 | 20 | 20 | 19 | (2.2) | 22 | 24 | 23 | 23 | 26 | 29 | (5.0) |
| Generic medicines | 194 | 201 | 197 | 207 | 215 | 212 | (1.8) | 68 | 73 | 81 | 76 | 82 | 94 | (6.7) |
| J (antiinfectives for systemic use) | | | | | | | |  |  |  |  |  |  |  |
| Single-source | 329 | 321 | 232 | 233 | 223 | 211 | (-8.5) | 478 | 482 | 484 | 413 | 421 | 440 | (-1.6) |
| Off-patent originators | 78 | 77 | 79 | 74 | 74 | 73 | (-1.3) | 240 | 88 | 93 | 78 | 81 | 103 | (-15.6) |
| Generic medicines | 2,754 | 2,915 | 3,027 | 3,314 | 3,185 | 3,009 | (1.8) | 1,200 | 1,376 | 1,335 | 1,158 | 1,140 | 1,285 | (1.4) |
| L (antineoplastic and immunomodulating agents) | | | | | | | |  |  |  |  |  |  |  |
| Single-source | 279 | 307 | 289 | 297 | 301 | 305 | (1.8) | 805 | 1,099 | 1,329 | 1,529 | 1,757 | 1,985 | (19.8) |
| Off-patent originators | 60 | 63 | 74 | 72 | 84 | 81 | (6.2) | 129 | 133 | 155 | 144 | 167 | 177 | (6.5) |
| Generic medicines | 565 | 553 | 499 | 505 | 482 | 474 | (-3.5) | 626 | 654 | 692 | 751 | 802 | 854 | (6.4) |
| M (musculo-skeletal system) | | | | | | | |  |  |  |  |  |  |  |
| Single-source | 184 | 179 | 134 | 127 | 120 | 121 | (-8.0) | 266 | 290 | 346 | 377 | 424 | 480 | (12.6) |
| Off-patent originators | 27 | 29 | 36 | 36 | 39 | 34 | (4.7) | 8 | 9 | 12 | 13 | 45 | 45 | (39.9) |
| Generic medicines | 1,732 | 1,759 | 1,796 | 1,962 | 1,885 | 1,842 | (1.2) | 650 | 694 | 712 | 712 | 714 | 794 | (4.1) |
| N (nervous system) | | | | | | | |  |  |  |  |  |  |  |
| Single-source | 469 | 475 | 440 | 443 | 435 | 422 | (-2.1) | 587 | 612 | 665 | 676 | 615 | 624 | (1.2) |
| Off-patent originators | 72 | 69 | 75 | 67 | 69 | 70 | (-0.6) | 53 | 55 | 62 | 55 | 46 | 62 | (3.3) |
| Generic medicines | 2,260 | 2,429 | 2,704 | 3,196 | 3,184 | 3,076 | (6.4) | 997 | 1,133 | 1,300 | 1,458 | 1,705 | 1,798 | (12.5) |
| P (antiparasitic products, insecticides and repellents) | | | | | | | |  |  |  |  |  |  |  |
| Single-source | 18 | 18 | 13 | 11 | 9 | 9 | (-12.9) | 4 | 4 | 4 | 4 | 4 | 4 | (2.4) |
| Off-patent originators | 4 | 5 | 4 | 3 | 4 | 4 | (0.0) | 0 | 0 | 0 | 0 | 0 | 0 | (-3.7) |
| Generic medicines | 27 | 25 | 24 | 28 | 25 | 25 | (-1.5) | 7 | 7 | 7 | 7 | 7 | 7 | (-0.4) |
| R (respiratory system) | | | | | | | |  |  |  |  |  |  |  |
| Single-source | 297 | 302 | 223 | 219 | 222 | 216 | (-6.2) | 309 | 314 | 314 | 264 | 263 | 362 | (3.2) |
| Off-patent originators | 31 | 30 | 43 | 38 | 38 | 41 | (5.8) | 6 | 4 | 15 | 12 | 9 | 16 | (23.7) |
| Generic medicines | 1,495 | 1,598 | 1,672 | 1,827 | 1,781 | 1,714 | (2.8) | 426 | 488 | 505 | 393 | 380 | 600 | (7.1) |
| S (sensory organs) | | | | | | | |  |  |  |  |  |  |  |
| Single-source | 370 | 369 | 265 | 248 | 215 | 205 | (-11.1) | 202 | 222 | 218 | 234 | 258 | 279 | (6.6) |
| Off-patent originators | 16 | 19 | 40 | 42 | 47 | 51 | (26.1) | 6 | 6 | 37 | 25 | 30 | 32 | (42.2) |
| Generic medicines | 776 | 948 | 1,034 | 1,227 | 1,143 | 1,065 | (6.5) | 286 | 318 | 401 | 433 | 476 | 528 | (13.0) |
| V (various) | | | | | | | |  |  |  |  |  |  |  |
| Single-source | 268 | 259 | 221 | 196 | 179 | 172 | (-8.5) | 229 | 234 | 257 | 240 | 262 | 265 | (3.0) |
| Off-patent originators | 65 | 63 | 64 | 65 | 77 | 75 | (2.9) | 39 | 40 | 50 | 57 | 77 | 77 | (14.7) |
| Generic medicines | 456 | 461 | 413 | 399 | 360 | 332 | (-6.1) | 262 | 281 | 297 | 318 | 341 | 355 | (6.2) |

AIGR: Average Annual Growth Rate (%)


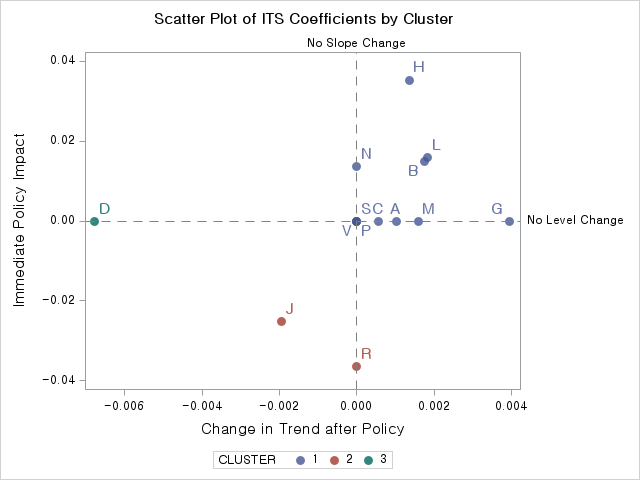


Supplementary Figure 1. Scatter plot of standardized interrupted time series (ITS) parameter estimates by cluster

The x-axis represents the change in trend after policy implementation, and the y-axis represents the immediate policy impact (level change at implementation). ATC classes are grouped into clusters based on similarity in these parameter estimates, illustrating heterogeneous response patterns across therapeutic categories following the tiered pricing policy.

A (alimentary tract and metabolism), B (blood and blood forming organs), C (cardiovascular system), D (dermatologicals), G (genito-urinary system and sex hormones), H (systemic hormonal preparations, excl. sex hormones and insulins), J (antiinfectives for systemic use), L (antineoplastic and immunomodulating agents), M (musculo-skeletal system), N (nervous system), P (antiparasitic products, insecticides and repellents), R (respiratory system), S (sensory organs), V (various)


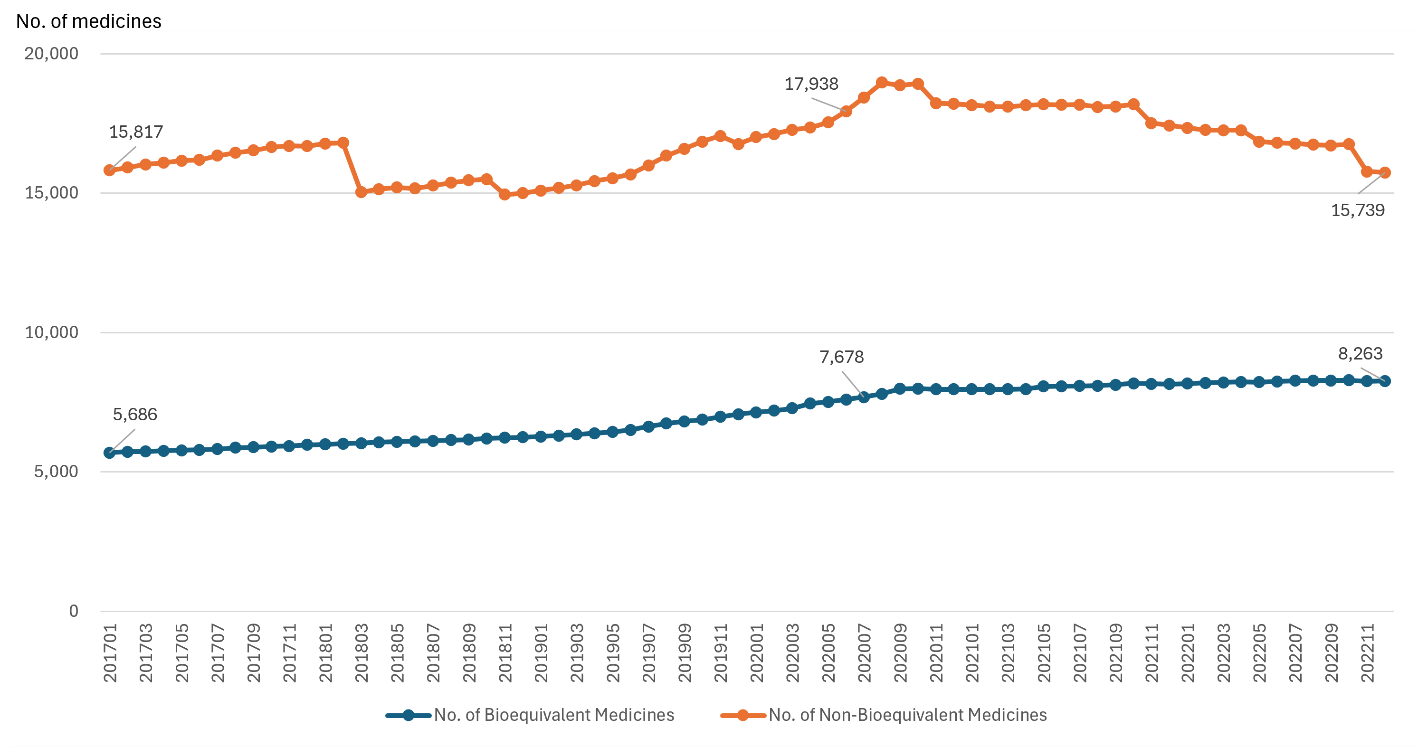


Supplementary Figure 2. Trend in the number of bioequivalent medicines after the new policy of generic medicines
